# Environmental risk factors provoke new thinking for prevention and treatment of dementia with Lewy bodies

**DOI:** 10.1016/j.heliyon.2024.e30175

**Published:** 2024-04-26

**Authors:** Dinghao An, Yun Xu

**Affiliations:** a Department of Neurology, Nanjing Drum Tower Hospital, Chinese Academy of Medical Sciences & Peking Union Medical College, Nanjing, China; b Department of Neurology, Nanjing Drum Tower Hospital, Affiliated Hospital of Medical School, Nanjing University, Nanjing, China; c State Key Laboratory of Pharmaceutical Biotechnology and Institute of Translational Medicine for Brain Critical Diseases, Nanjing University, Nanjing, China; d Jiangsu Key Laboratory for Molecular Medicine, Medical School of Nanjing University, Nanjing, China; e Nanjing Neurology Clinical Medical Center, Nanjing, China

**Keywords:** Dementia with Lewy bodies, Environmental factors, A-synuclein

## Abstract

In recent years, environmental factors have received attention in the pathogenesis of neurodegenerative diseases. Other than genetic factors, the identification of environmental factors and modifiable risk factors may create opportunities to delay the onset or slow the progression of Lewy body disease. Researchers have made significant progress in understanding environmental and modifiable risk factors over the past 30 years. To date, despite the increasing number of articles assessing risk factors for Lewy body disease, few reviews have focused on their role in its onset. In this review, we reviewed the literature investigating the relationship between Lewy body disease and several environmental and other modifiable factors. We found that some air pollutants, exposure to some metals, and infection with some microorganisms may increase the risk of Lewy body disease. Coffee intake and the Mediterranean diet are protective factors. However, it is puzzling that low educational levels and smoking may have some protective effects. In addition, we proposed specific protocols for subsequent research directions on risk factors for neurodegenerative diseases and improved methods. By conducting additional case-control studies, we could explore the role of these factors in the etiopathogenesis of Lewy body disease, establishing a foundation for strategies aimed at preventing and reducing the onset and burden of the disease.

## Highlights

• Coffee intake and the Mediterranean diet are low risk factors associated with Lewy body dementia.
• Infection with some pathogenic organisms may be associated with an increased risk of Lewy body dementia.
• Low educational level and heavy smoking may have some protective effect in Lewy body dementia.
• Air pollution exposure may act as trigger factor in Lewy body dementia.
• Some heavy metals may be significantly relevant to the onset of Lewy body dementia by increase the aggregation of a-synuclein.

## 1 Introduction

Dementia with Lewy bodies (DLB) is a neurodegenerative disease that is clinically characterized by fluctuating cognitive impairment, parkinsonism, and vivid visual hallucinations. It is mostly sporadic, and the etiology and risk factors of DLB have not been fully clarified. Neuron damage in DLB is suspected to be caused by a variety of factors, including genetic, lifestyle, and environmental factors. The substances in Lewy bodies are a-synuclein and ubiquitin, and abnormal protein deposition may lead to neuronal dysfunction and apoptosis. Although genetic factors have been identified, only a small proportion of the overall risk of neurodegenerative diseases can be attributed to genetic alterations. Moreover, the pathophysiological characteristics of DLB are similar to Parkinson's disease dementia (PDD), both belonging to Lewy body dementia (LBD), and Parkinson's disease dementia is closely related to environmental factors. Therefore, some experts believe that environmental factors seem to play an important role in the risk of DLB [1]. Studies have shown that smoking and low education are not risk factors for DLB, while depression and low caffeine intake are strong risk factors for patients with DLB [2]. So we also consider DLB as a disease mediated by a combination of genetic susceptibility and environmental factors. DLB, a major problem in neurology, has been studied throughout the world to find new ways to reduce the risk factors and slow its progression through interventions. There are many reports on risk factors for DLB based on studies of univariate analysis and multivariate analysis, but few reviews have specifically and systematically focused on the role of various environmental risk factors in the occurrence or progression of DLB. Here, we reviewed multiple environmental factors and modifiable lifestyle risk factors for DLB, including eating habits, metal and chemical exposure, infection, education, smoking and alcohol abuse, and psychosocial factors.

## 2 Database and search method

In this paper, we analyzed and integrated papers from six databases: PubMed, Cochrane, Google Scholar, Embase, Scopus, and Clinical Trials gov ranging from January 1st, 1984 to October 1st, 2023. We prioritized papers from the past 5 years. The search results were included according to the title and abstract, and the papers were reviewed in full text. The keywords used for searching included two groups, the first one includes Lewy body dementia, dementia with Lewy bodies, Lewy body disease, LBD, DLB, and a-synuclein. While the second one includes risk factors, associated factors, environmental factors, nutrients, diet, nutritional status, trace elements, metals, heavy metals, toxicants, climatic factors, climate change, air pollution, physical factors, heat, ultraviolet light, magnetic radiation, noise, microorganisms, bacteria, virus, lifestyle, smoking, alcohol, psychosocial status, depression, anxiety, panic disorders, education. These keywords were used in conjunction with the Boolean operators AND and OR. The search string for the PubMed database was ((“Lewy body dementia” [Mesh]) OR (LBD)) AND (“risk factors” [Mesh]), ((“dementia with Lewy bodies” [Mesh] OR (DLB)) AND (“risk factors” [Mesh]), ((“Lewy body disease” [Mesh] OR (LBD)) AND (“risk factors” [Mesh]), ((“a-synuclein” [Mesh] OR (alpha-synuclein)) AND (“risk factors” [Mesh]). Then we replaced “risk factors” with the keywords from the second group for comprehensive retrieval. We searched four times in total for each keyword. After strict screening, we also examined the reference catalogs of relevant studies and previous reviews.

## 3 Diet and nutrition

### 3.1 Diet

It is well-known that overeating and malnutrition can have different adverse effects on the body, such as fussy eating, improper food matching and even chemical reactions between foods may increase the risk of diseases. The Mediterranean diet (MeDi) and Dietary Approaches to Stop Hypertension (DASH) diet have been shown to protect various organs throughout the body. This diet consists of mainly fruits, vegetables, poultry, seafood, whole grains, beans, nuts, and seeds. A new longitudinal study suggests that the Mediterranean diet is associated with a lower incidence of prodromal LBD. This diet may protect from alpha-synuclein aggregation and early neuronal degeneration, presumably in the gut and several areas in the brain. Moreover, the MeDi components (such as carotenoids, tocopherols, phenolics, probiotics, fibers, phytoestrogens, etc.) foods and the whole MeDi pattern may have other effects including antioxidant, and anti-inflammatory properties [3].

As mentioned earlier, low caffeine intake is a strong risk factor for patients with DLB. In addition to a substance found in coffee, eicosanoyl-5-hydroxytryptamide can lead to reduced aggregation of phosphorylated a-synuclein, preserve neuronal integrity and function, reduce neuroinflammation, and improve behavioral performance, thus acting as a low-risk factor for DLB, with a synergistic effect with caffeine [4].

In addition, the fruits of the Annonaceae family (soursop, sweetsop, and custard apple) which are commonly eaten in Guam are found to be the cause of Guadeloupean parkinsonism. The toxins of this family are incriminated in the cause of Guadeloupean parkinsonism, which contains three neurotoxins, including benzyltetraisoquinoline reticuline, tetrahydroberberine coreximine, and annonacin [5]. Although they can cause the accumulation of a-synuclein, the core substance of DLB remains unknown and has not been further studied since then.

Some researchers in Japan have confirmed the effect of plants on the occurrence and development study of DLB. Spearmint is a kind of plant of the mint family, and the spearmint extract and rosmarinic acid (the major component of SME) have effects on the amyloid fibril formation of a-synuclein, they can disassemble a-synuclein into non-toxic part [6].In addition, it has been pointed out that inorganic phosphate may be the rate-limiting factor for tau hyperphosphorylation in LBD [7].However, no clinical trials ever tested hypophosphatemic diets to treat and prevent DLB [8].

### 3.2 Nutrition

In addition, the nutritional status of patients is also widely recognized as having an integral role in the development of diseases. Adequate nutrition is necessary to ensure the normal function and repair of organs and systems in people with dementia [9]. In Japanese elderly people with DLB, the reported incidence of malnutrition varies from 13 % to 57 % [10].Malnutrition is associated with faster functional decline over five years, poorer prognosis, more comorbidity, weaker constitution, higher mortality, and greater cognitive impairment [11]. This may occur through various mechanisms, such as decreased mobility, muscle wasting, decreased immunity, fatigue, drowsiness, depressed mood insomnia, etc. However, it is questionable that studies have shown that malnutrition is associated only with faster functional loss and not with cognitive decline in elderly people with dementia [12], which conflicts with previous studies showing that patients with DLB developed malnutrition status at baseline and during follow-up after micronutritional assessment [13]. Studies have also demonstrated that LBD patients have a higher risk of malnutrition compared to other dementia [14]. Patients with long-term weakness, dementia, aging anorexia, anxiety and depression, various infections, anticholinergic and sedative drugs, and other factors will reduce food intake, reduce salivary secretion, and aggravate dysphagia, it's a vicious circle. Therefore, researchers and expert groups need to discuss whether malnutrition in patients with DLB is a cause or an outcome. There is still a large gap in exploring the in-depth mechanism of the effects of nutrients and diet style on DLB directly and indirectly. Therefore, it needs a large observational study based on a large sample and follow-up analysis to further debate whether nutritional status will have a critical impact on the occurrence and development of DLB.

In addition, the rise of lipidomics in recent years has provided certain ideas for the onset and progression of central nervous system diseases. Since Lewy bodies not only enrich in a-synuclein but also enrich in lipids and other substances, some experts proposed that LBD is a lipidosis. There is already evidence to suggest that hyperlipidemia can increase the risk of LBD, and several genes related to lipid metabolism have been identified as genetic risk factors for the onset and progression of Parkinson's disease [15]. A study found that changing the concentration of certain lipids in PD models, such as the synthesis of phosphatidylethanolamine, can lead to the accumulation of a-synuclein, resulting in endoplasmic reticulum stress and mitochondrial dysfunction [16].It can also cause saturation of neurons and disrupt their cellular mechanisms and functions, leading to inflammatory reactions and damaging neural circuits [17]. In addition, certain lipid metabolism pathways are closely related to the accumulation of a-synuclein, and lipids seem to be a key contributor to the toxicity of a-synuclein. Conversely, the physiological level of a-synuclein maintains lipid homeostasis. The occurrence of hyperlipidemia is largely caused by abnormal dietary structure and excessive nutritional status. Therefore, we speculate that a low-fat or low-lipid-conversion diet may also reduce the occurrence and development of DLB to some extent.

## 4 Intoxication and pollution

### 4.1 Trace elements and heavy metals

Environmental toxicants, particularly heavy metals, have been identified as modifiable risk factors for cognitive impairment. Identifying and reducing contact is therefore critical. In recent years, research in this area has gradually increased, providing evidence for the prevention of diseases. Metals include highly toxic metals and metalloids, which are characterized by high toxic capacity even at low concentrations [18]. They are widely distributed in the environment and are used in many fields. Some metals such as iron, copper, zinc, or calcium are known as essential elements because they are involved in various biological processes, such as neurotransmitter synthesis, neuronal myelination, mitochondrial function, neuronal development, cellular homeostasis, antioxidant process, and DNA repair [[19], [20], [21]]. Experiment showed that the combination of CSF-Mg and CSF-Ca could distinguish DLB from AD with 93 % sensitivity and 85 % specificity [22].Besides, calcium, magnesium, and copper are increased in DLB patients [22]. Arsenic can cause learning and memory impairment and emotional deficits [23]. Regarding cadmium, it can be transported directly across the blood-brain barrier and eventually accumulate in the brain to activate signaling pathways involved in inflammation, oxidative stress, and neuronal apoptosis [24]. It has been shown that crosstalk of metal homeostasis can play a role in the pathophysiology of a variety of neurodegenerative diseases, including DLB [25]. The relationship between metals and LBD and a-synuclein has been extensively investigated [26]. The oligomerization and fibrillation of a-synuclein have been implicated in the etiology of DLB, and the precursors of fibrils, oligomers of a-synuclein, might be more toxic than fibrils [27,28]. V.N.Uversky et al. once reported that a-synuclein could oligomerize in the presence of different metals including Al, Cd, Cu, and Fe [[29], [30], [31], [32]].

Mn-induced neurological condition is characterized by motor skills and cognitive impairment [33,34]. Degeneration areas include the medial globus pallidus, cerebellum, pons, substantia nigra, reticularis, red nucleus, thalamus, cortex, and spinal cord [[35], [36], [37]]. In rare cases, it may involve the caudate and putamen [38]. a-synuclein is overexpressed and forms oligomer in cells exposed to manganese [39,40], and thus may be relevant to the mechanism of DLB [41].Serum samples showed elevated levels of misfolded a-synuclein in exosomes [42].

Numerous studies have shown LBD patients have increased iron concentrations in substantia nigra, putamen, and globus pallidus [43]. For one thing, a-synuclein promotes iron accumulation in neurons, and for another, iron promotes oligomerization of a-synuclein, showing extensive and widespread tendency [44]. It is a vicious cycle, associated with DLB [45,46].

For zinc, this is a controversial element, which has both toxic and nutritional effects. It has been demonstrated that zinc is associated with depressive symptoms in DLB [47,48]. In addition, in tissues from patients who died due to DLB, it was suggested that zinc ions enter synaptic vesicles to function as long-term modulators of synaptic activity [49,50]. However, DLB was not significantly associated with zinc in a 2014 study [51], so whether zinc has a role in regulating neurodegenerative diseases remains controversial. Copper exposure appears to accelerate the progression of a-synuclein pathology [52].

In LBD, Cu and Cu transporter 1 levels were significantly reduced in vulnerable substantia nigra and locus coeruleus [53]. Fredrik Boström et al. observed elevations of CSF-Cu to be associated with the neurodegenerative process in DLB [22].

The toxicity of Pb includes memory loss, decreased neural signals, and decreased learning ability [54].In addition, lead can also cause a-synuclein accumulation [55], which may act as a risk factor [56,57].

Aluminum has been linked to the etiology of neurodegenerative conditions [58]. However, there is a paucity of research literature on aluminum and DLB directly, and it doesn't promote the aggregation and oligomerization of a-synuclein.

Mercury has been reported to cause cognitive impairment in the brain [59]. Moreover, mercury has multiple toxic effects on the central nervous system. Mercury causes degradation of spatial cognition in a model [60]and can induce a-synuclein aggregation and often co-localized with aggregated a-synuclein [61], therefore being a risk factor to trigger DLB.

Arsenic can cause degeneration and death of neurons [62]. Repeated arsenic exposure for 28 days was associated with LBD [63], and like aluminum, arsenic poisoning did not trigger LBD by promoting a-synuclein aggregation and oligomerization. However, it can lead to elemental imbalance and act as a risk factor for developing neurodegenerative diseases [64].

However, direct estimation of exposure to toxic substances from epidemiological data is difficult. As for trace elements or metals, previous studies did not perform the concentration titration method to carry out the experiments, and due to multiple confounders, it is easy to produce completely contradictory conclusions. And we do not know the exact concentration or the range of one metal. In addition, DLB does not affect the entire brain in the ultra-early stages of the disease, and it is difficult for us to detect toxic substances for only a small proportion of structures in the brain. Therefore, it's important to have elemental bioimaging of potentially toxic elements at the cellular level to estimate the toxicant burden in the brain.

### 4.2 Chemical toxicants

Chemical toxicants in environmental media such as water, food, air, and soil that we contact with every day, even at relatively low concentrations, can lead to serious diseases due to exposure to complex mixtures. Non-targeted metabolomics experiments have shown the presence of phthalates in the cerebrospinal fluid of patients with DLB [1]. Phthalates are synthetic industrial chemicals extracted from phthalic acid. They were first introduced as plasticizers in the 1920s and are widely used in a variety of fields. Since then, the first case of DLB has been described in the 1960s and 1970s. These observations suggest that phthalates are likely to be involved in the occurrence of DLB.

In addition, some plants in nature also affect a-synuclein aggregation. Saffron, the stigma of Crocus sativus Linné (Iridaceae family), has been known to inhibit aggregation of a-synuclein [65] water-soluble spearmint extract and rosmarinic acid can inhibit a-synuclein, tau formation, which is associated with DLB [6]. These Annonaceae plants contain insecticidal substances, including water-soluble alkaloids: benzyl-tetra-hydro-isoquinolines and isoquinoline derivates, tetra-hydro-protoberberines, and a new class of polyketides: acetogenins. Annonacin, a selective mitochondrial complex I inhibitor, is included in the fruits and leaves of A. spinosum as a possible cause of LBD [66]. Cycad are seed foods for some mammals, containing the compound β-N-methylamine-l-alanine. When humans consume mammals, due to the bioaccumulation effect, this may lead to DLB [67]. Many extracts can also reduce the aggregation of a-synuclein in a transgenic model expressing “human” a-synuclein worms, including Mucuna pruriens seed extract, red seaweed Chondrus crispus, Dioscorea alata L. tubers, Sorbus alnifolia and Holothuria leucospilota [68]. According to a new study, two polyphenols from olive oil, hydroxytyrosol, and oleuropein aglycone, attenuated the a-synuclein-induced locomotion impairments [69]. This is worthy of being further studied in DLB. Some azo dyes like Sunset Yellow, Congo Red, and Allura Red can induce tau aggregation [70]. However, no studies are reporting its association with the onset of DLB except for PD [71]. Vinyl chloride and Trichloroethylene are halogenated solvents widely used as a degreaser and have been identified as a risk factor for PD. They are mainly released into the air, soil, and groundwater. After long-term exposure, not only does a-synuclein accumulate, but it also spreads [72]. Interestingly, symptoms of the patients include cognitive impairment, dementia, and hallucinations. This is likely due to diagnostic bias, with DLB patients included. Therefore, we speculate vinyl chloride is also a risk factor for the occurrence of DLB. Because most positive research was not carried out in DLB patients and is mostly based on the hypothesis of a-synuclein, validation needs to be done through mouse models after exposures to the toxicants to reduce the confounders as much as possible.

Many studies have found an increased prevalence of cognitive and psychomotor dysfunction in individuals chronically exposed to pesticides. The prevalence of dementia has risen dramatically in recent decades, roughly after increasing pesticide use decades ago [73]. A previous American Prospective cohort study based on people more than 65 years old without dementia noted an increased risk of all-cause dementia following 3–10 years because of relevant occupational exposures [74].Pesticide exposure has repeatedly been identified as a risk factor for neurodegenerative diseases [75], but only some specific pesticides [76].MPTP, paraquat, and rotenone can lead to acute parkinsonism [77]. Given the similar pathophysiological mechanisms and the clinical overlap between PD and DLB, it has been suggested that pesticide exposure may also be involved in the pathogenesis of DLB [78]. And it has been shown that certain pesticides significantly stimulate the rate of formation of a-synuclein fibrils, including rotenone, DDT, 2,4-D, dieldrin, diethyldithiocarbamate, paraquat, maneb, trifluralin, parathion, and imidazoldinethione. However, some pesticides have no significant effect on the kinetics of a-synuclein fibrillation, including iprodione, glyphosate, methomyl, thiuram, mevinphos, carbaryl, alachlor, thiobencarb [79]. Organophosphates have also been shown to cause microtubule disturbance and tau hyperphosphorylation, a hallmark of LBD [80]. Many studies have shown that there is a decline in cognitive function [[81], [82], [83], [84], [85]] and Parkinson-like symptoms [86] in people who have been exposed to pesticides for a long time. Most of the cognitive impairments in these studies were declines in attention, processing speed, visual construction, and orientation, which are characteristic of cognitive impairment in DLB. Subsequent neurologists should also consider pesticide exposure when asking patients, and the expert community should reach a new consensus to determine exposure times and expand animal experiments. Correspondingly, more prospective cohorts and observational studies are needed for the most harmful specific toxicants. In any case, minimizing pesticide exposure is a wise precaution.

### 4.3 Air pollution

Air pollution is on the list of potential risk factors for dementia, even at relatively low levels [87,88]. And a longitudinal study suggests that this includes LBD [89]. An epidemiological association has recently been made between long-term air pollution and dementia incidence in the Swedish population [89]. Prolonged exposure to nitrous oxide (N2O) may be a risk factor for LBD [90]. The evidence of a causal relationship between fine particulate matter and dementia is encouraging [91]. The pollutants, especially PM2.5, and O3 association are highly acknowledged, while NO, PM10, and NO2 studies and dementia associations are still contradictory and need to be debated [92]. The possible reason may be due to genetic factors like race and ethnic residence influence. Since the range of dementia cases reported in cohorts is wide, an accurate allocation of dementia is difficult. The clarified sample size of DLB is small, and a limitation of cohort study may be that pollutant concentrations may be more likely to change in long-term studies, as well as the influence of confounding factors. In addition, air pollutants per se and physical factors are also correlated.

## 5 Climatic and physical factors

### 5.1 Climate and heat

Recently literature suggested a significant association between dementia and increased vulnerability to extreme heat [93,94]. Climatic factors have also been shown to have some effect on neurodegenerative diseases [95].Environmental exposures and adverse climates may trigger inflammation in neurodegenerative diseases, and studies have reported an increased incidence of DLB [96,97] due to climate change. And a-synuclein abnormally accumulated subsequently in DLB, leading to apoptosis, especially HSP90 [98]. In addition, elevated temperatures may increase the volatility of contaminants in soil and water [99]. Heat exposure can cause neuronal RNA oxidation [100], which is a prominent feature of LBD. Increased precipitation and extreme weather events may lead to spillage of contaminated land, which may lead to the reflow of contaminants in sediments and freshwater contamination. Other reasons associated with climate change include active viruses, which have direct and indirect neurological effects. In the fifth part, we will discuss the impact of viral infections on neurodegenerative diseases in detail, especially LBD. Climate change can also affect emotional responsiveness and cognitive systems [101]. Human behavior such as burning, and industrial pollutants can lead to invisible pollutants and neurotoxins in the atmosphere. Their deposition on food and water impacts the nervous system potentially [102]. Changing climatic conditions promotes the spread of pathogens, exacerbates environmental pollution, increases the risk of exposure to harmful neurotoxicants, and may also directly perturb brain physiology.

### 5.2 Ultraviolet light

In addition, photoperiod is also an environmental part, which plays a role in neurodegenerative diseases. Aumann et al. showed the first evidence of an association between photoperiod and the number of Dopaminergic (DA) neurons in the human midbrain [103]. Dityrosine (DiY) is a protein modification product of oxidative stress, and DiY modification of a-synuclein can prevent its aggregation in the absence or low aggregation under UV irradiation. More importantly, a-synuclein is very sensitive to UV-induced DiY formation. However, as aggregation proceeds, DiY formation may shift from an aggregation suppressor to a promoter [104].

### 5.3 Magnetic radiation

Moreover, a meta-analysis of occupational electromagnetic fields and neurodegenerative diseases showed that occupational electromagnetic field exposure was weakly associated with AD, but not with other dementias [105]. However, misclassification of disease and imprecise exposure assessment caused much bias.

### 5.4 Noise

For noises, it has been suggested that noise exposure may interact synergistically with air pollution, leading to a stronger decline in cognitive performance [106]. But this is rebutted by new studies that it’s not significantly associated. The researchers found that exposure to noise levels(24h > 55 dB) had no significant effect on dementia risk [107].In addition, our comprehensive search did not find any studies on sunlight exposure and the risk of DLB.

## 6 Infection and micro-organisms

It has long been suspected that infectious factors are associated with the Prion-like propagation of a-synucleinopathies. Currently, interest in this hypothesis seems to be renewed, and viral infections are increasingly recognized as a risk factor for neurodegenerative diseases, especially in the study of lentiviruses. The first reason is the potential antimicrobial role of a-synuclein [108]. And the second is the potential entry of pathogens regarding early symptoms of a-synucleinopathies [109]. Neuroinvasive viral infections may be epidemiologically and mechanically associated with neurodegeneration [110]. For example, a-synuclein can act as a viral restriction factor to inhibit West Nile Virus(WNV) infection [111]. It has been suggested that anti-EBV antibodies cross-react with a-synuclein, and in patients with PDD [112] and so does HSV1 [113]. Another study demonstrated that Coxsackie virus B3 infection also induced the formation of a-synuclein in neurons [114]. a-synuclein aggregation has been successfully induced in models and rodents in vitro, associated with different pathogens (including H5N1 Virus, H1N1 Virus, WNV, Western Equine Encephalitis Virus, Capreomycin-producing E. coli, Lipopolysaccharide-producing Proteus) [[115], [116], [117], [118], [119]]. A clinical case report of probable DLB is illuminating, that's a patient who presented with symptoms of DLB immediately following WNV encephalitis [120]. A recent experiment shows that Lewy bodies formed after vaccination of SARS-CoV-2 [121,122]. In addition, norovirus has also been isolated from patients with DLB, indicating active infection in the colon, secretion of a-synuclein by the intestine, and researchers found co-localization of norovirus and a-synuclein in different regions of the patient's brain, which is positively correlated with viral persistence, so maybe norovirus infection can migrate from the intestinal mucosa to the central nervous system through the autonomic nervous system and contribute to the development of disease [123,124]. Influenza virus infection also induces Parkinsonian symptoms and increases phosphorylation and aggregation of a-synuclein. Repeated viral infections may induce a-synuclein expression or/and a-synuclein aggregation, and chronic viral infections induce further inflammation, which may trigger and contribute to the occurrence of DLB. In addition, it has been suggested that Nocardia asteroides [125] and Borrelia burgdorferi are also involved in the occurrence of DLB, which has a strong correlation [126], but the sample size is too small. In another cross-sectional study including 37 patients with DLB, a history of systemic infection treated with antibiotics was significantly associated with dementia onset at an older age [127]. In summary, infection with foreign microorganisms is likely to be a trigger for inducing the onset of DLB. However, various studies remain limited, firstly, they have been intensively studied based on the mechanistic hypothesis of a-synuclein. Secondly, it is unclear whether a-synuclein aggregates trigger the degenerative process of DLB, whether they are a collateral effect as a result or even a protective mechanism to prevent loss of proteostasis due to dysfunction of the protein degradation system.

## 7 Lifestyle

### 7.1 Smoking

Tobacco has harmful effects on almost all diseases, as well as in the nervous system, and its effects on CNS include decreased sensitivity and precision of neuromuscular responses, insufficient tissue oxygen, neuronal necrosis, and apoptosis, increased oxidative stress and cerebrovascular injury, etc. It's also harmful in neurodegenerative diseases, and most studies have shown that smoking is a risk factor for cognitive decline, such as increasing the incidence of AD, while studies in LBD have shown no significant association [128],which is consistent with a Norwegian study [129]. However, it is interesting that lifetime heavy smoking (>50 pack-years) is associated with a significantly lower risk of LBD in a community-based study, and this apparent protective effect was only observed in heavy smokers, suggesting a possible exposure-response relationship [130], which is consistent with a recent study [131].In addition, a study including 200 patients(87 LBD) found that rapid cognitive decline did not occur in the LBD group, which shows that smoking did not accelerate the progress of LBD [129].We hypothesize the apparent protective effects of smoking may be a result of survivor bias and need to be further studied. We cannot simply think heavy smoking acts as a protector for DLB. Although smoking is too toxic to recommend as a treatment, these findings, if confirmed, may guide future strategies to suppress DLB by other particles in cigarettes. We still recommend ingredients in the tobacco should be studied.

### 7.2 Alcohol

In addition, it is well-known that alcohol triggers paralytic effects on the vestibulo-cerebellar system, neuronal death, and withdrawal reaction based on the limbic system, deficiency of vitamin B. High correlations between alcohol and cognitive function have also been shown in DLB [127]. Excessive alcohol consumption may increase the risk of cognitive impairment, but low to moderate alcohol intake may prevent cognitive decline and dementia, these findings should be interpreted with caution due to different methods and lacking standardized definitions. However, it is contradictory that alcohol is not a risk factor for DLB suggested by a recent cohort study [132]. Some kinds of alcohol have positive cognitive effects, we think it may not be ethanol itself, but other substances in alcohol exert influence on the good side, which need to be confirmed further.

## 8 Psychosocial status and education

### 8.1 Psychosocial status

Socioeconomic and personality characteristics are factors that cannot be ignored for the development of diseases. It has also been suggested that patients with DLB have significantly reduced participation in social activities, and in a cross-sectional study of patients with newly diagnosed DLB, most of the patients were living alone and had little contact with the outside world except for daily care needs [[133], [134], [135]]. An Internet survey was conducted by the Lewy Body Dementia Association on caregivers who had been caring for people with DLB for a long time, 384 in total, and the outcome was consistent with previous research [136]. In an in-depth interview of five patients with DLB in Sweden, patients indicated an inability to participate in social situations, embarrassed with falling, fear of dependence on others, and loss of confidence in going outside [137], resulting in a sense of worthless, reduced self-esteem, withdrawal behavior.

In addition, anxiety and depression have also been shown to be strong risk factors and common prodromal neuropsychiatric symptoms in patients with DLB. Baseline depression scores were higher in DLB, and the two exacerbate each other, aggravating the progression of the disease [48,138,139]. which may be due to problems in the regulation and control of the ponto-limbic emotional circuit. Especially anxiety and depression, they may effectively reflect the preclinical lesions in patients with LBD. We hypothesize psychosocial mechanism may be related to abnormal regulation of the hypothalamic pituitary adrenal axis; Excessive activity of the autonomic nervous system; and immune system disorders, at the same time, it is strengthened by special environments, causing changes in brain networks and neurotransmitters, leading to the occurrence of neurological diseases. The causal relation between the two needs to be further validated. Without a doubt, further research is also needed to determine the duration of anxiety and depression before LBD diagnosis and to identify features that can be used to diagnose in the early stage.

As for marriage and childbearing, having more than one child in a father is a risk factor for Parkinson's disease, but does not increase the risk of DLB. However, earlier dementia onset in married people may be due to earlier detection of functional impairment through their partners.

### 8.2 Education

Although the aging rate of the highly educated brain is slower than that of the less educated, maybe this is because the structural connectivity of neurons is denser during the continuous generation of new synapses, thus forming more complicated neural networks. Therefore, it has been shown in dementia-related diseases that both high education and continuous learning and training are nootropic factors, and such patients have a greatly reduced risk of cognitive impairment. But interestingly, this does not apply in most studies on DLB. A previous study speculated that receiving less than 5 years of formal education may be a risk factor for DLB compared to individuals without cognitive impairment [140]. However, paradoxically, researchers found that higher education was more common in cases with a clinical diagnosis of DLB (n = 147) [2], and a cohort study from Norway included a limited sample size, mainly highly educated participants(N = 87). Increased education as a risk factor for earlier LBD onset was unexpected and contrasted with the notion of cognitive reserve [132]. High educational level is considered a protective factor against PDD, however, it is associated with a higher risk of DLB [141,142] and it has been shown that a high educational level does not distinguish LBD from the rate of cognitive decline in controls [143]. This suggests that low education is not a risk factor for the incidence of LBD, on the contrary, it could be a protective factor. We need to think rationally because higher education indeed can benefit us a lot and we'd better not give up the opportunity to accept education.

## 9 Conclusion and future directions

There is an increasing interest in identifying the risk factors associated with the occurrence and progression of DLB, intending to develop prevention or management strategies. Numerous studies have investigated environmental and modifiable risk factors for dementia with Lewy bodies. The evidence suggests that certain eating habits, such as consuming large amounts of Annonaceae fruits, exposure to certain metals and chemicals, and social stress, are high-risk factors for DLB. On the other hand, caffeine intake is a low-risk factor. However, it is puzzling that low educational levels and smoking may have a protective effect. Residential environment, climate, and other factors may also be associated with it, but due to the limitation of sample size, the establishment of a larger database to further validate is also required.

Future studies need to focus on modifiable factors of DLB, particularly the impact of any specific eating habits or environment. Although some associations between modifiable factors such as education, living environment, chemical toxins, and stress and DLB have been preliminarily confirmed, their specific role in the pathogenesis remains limited and needs to be further investigated. Therefore, future prospective research should ensure risk factors are monitored in DLB patients throughout the extended follow-up period. Moreover, little is known about protective factors at present. Cohort studies on prodromal DLB could explore this by prospectively recording lifestyle, dietary patterns, and other environmental risk factors before or early in the onset of cognitive decline.

In addition, it is increasingly recognized that gene-environment interactions are determinants of disease phenotypes, and currently, as genetic testing becomes more accessible, this allows us to identify high-risk individuals who may benefit most from interventions. Genes and prognostic models involved in the function of specific chemicals should be explored to find the sites of their function and related signal transduction pathways, thereby finding potential therapeutic targets and preventing the occurrence of DLB. However, there is no research on DLB patients with a history of long-lasting residential changes before the onset of the disease. For those descriptive studies with only one study having a significant result, diagnosis as well as survival bias may lead to false positive results. This requires us to conduct a high-quality study, a large-scale multicenter sample dataset. And careful history-taking with exposure factors is also needed. We all expect to find relevant risk factors that play a decisive role in the etiology and pathogenesis to conquer Lewy body dementia at the root cause. This review systematically lists reported risk and protective factors. And it may be valuable for preventing and treating DLB. We hope this review will shed light on subsequent studies based on patient recruitment.

## 10 Primary validation

Due to the limitations of observational and cohort studies, and multiple confounders in the previous research, we performed some validation. And because some research conclusions are inconsistent and the advantages of Mendelian Randomization, we used it to explore the genetic associations between different exposures and DLB. The datasets come from the open GWAS database. And because this paper is a review, so we only show the results here. Strangely, our results did not show any significant genetic correlation between DLB(ID: ebi-a-GCST90001390) and several exposures, like past tobacco smoking(ID: ukb-b-2134), previous alcohol drinker status(ID: ukb-a-227), years of education(ID: ebi-a-GCST90029013), social anxiety or social phobia(ID: ukb-*d*-20544_1), depression(ID: ebi-a-GCST90038650), anxiety(ID: ebi-a-GCST90038651), overall healthy diet(ID: ebi-a-GCST90096893), caffeine levels(ID: ebi-a-GCST90026134), malnutrition(ID: finn-b-E4_MALNUTRITION), iron(ID: ukb-b-20447), copper(ID: ieu-a-1073), zinc(ID: ieu-a-1079), calcium(ID: ukb-b-8951) and magnesium(ID: ukb-b-7372). Due to the data availability of other exposures, we only chose the above factors.

In short, for factors that are detrimental to our health, if they can be controlled and modified, we should try to avoid or correct them as early as possible, which may help in the onset or progression of the disease to some extent. Moreover, since some viruses do not have virus-related IgGs or hereditary substances in the body after recovery, Mendelian Randomization analysis requires SNPs with a history of virus infection or a new panel of toxic substances. The next step is to identify SNPs related to GWAS based on genetic susceptibility, such as some heavy metals, chemicals, and harmful gases, and then conduct further analysis on DLB.

## Confirmation of authorship

All authors have confirmed their authorship, their affiliations, their contributions and have approved its publication.

## Ethics declarations

Review and/or approval by an ethics committee and informed consent were not needed for this study because this review is a summary and analysis of existing literature, and it does not involve research on human subjects or specific experiments.

## Data availability statement

Data Availability No.

Sharing research data helps other researchers evaluate your findings, build on your work and to increase trust in your article. We encourage all our authors to make as much of their data publicly available as reasonably possible. Please note that your response to the following questions regarding the public data availability and the reasons for potentially not making data available will be available alongside your article upon publication. Has data associated with your study been deposited into a publicly available repository?

Please select why. Please note that this statement will be available All data has been included alongside your article upon publication. in article/supp. material/as follow-up to “Data Availability” referenced in article.

## CRediT authorship contribution statement

**Dinghao An:** Writing – original draft, Methodology, Formal analysis, Conceptualization, Investigation, Project administration, Writing – review & editing. **Yun Xu:** Writing – review & editing, Supervision, Conceptualization, Data curation, Formal analysis, Funding acquisition, Investigation, Methodology, Project administration, Resources.

## Declaration of competing interest

The authors declare that they have no known competing financial interests or personal relationships that could have appeared to influence the work reported in this paper.
